# Children, young people and the Commercial Determinants of Health

**DOI:** 10.1093/heapro/daad185

**Published:** 2024-01-31

**Authors:** Hannah Pitt, Simone McCarthy, Grace Arnot

**Affiliations:** Institute for Health Transformation, Faculty of Health, Deakin University, 1 Gheringhap Street, Geelong, Victoria 3220, Australia; Institute for Health Transformation, Faculty of Health, Deakin University, 1 Gheringhap Street, Geelong, Victoria 3220, Australia; Institute for Health Transformation, Faculty of Health, Deakin University, 1 Gheringhap Street, Geelong, Victoria 3220, Australia

**Keywords:** commercial determinants of health, children, young people, public health

## Abstract

The commercial determinants of health (CDoH) have a significant impact on the health and well-being of children and young people (subsequently referred to as young people). While most research has focused on the influence of harmful industry marketing on young people, more recent CDoH frameworks have emphasized that a range of commercial systems and practices may influence health and well-being. Focusing on the impact of traditional and digital media, contemporary marketing strategies and corporate production and consumption processes, the following article outlines the impact of the CDoH on the health and wellbeing of young people. The article also provides evidence about how young people conceptualize the impact of corporate actors on health, and their involvement in advocacy strategies to respond. The article recommends that when collaborating with young people to understand the impacts of and responses to the CDoH, we should seek to diversify investigations towards the impact of a range of corporate tactics, systems and structures, rather than simply focusing on the impacts of advertising. This should include considering areas and priorities that young people identify as areas for action and understanding why some young people are more vulnerable to commercial tactics than others. Youth are powerful allies in responding to the CDoH. Public health and health promotion stakeholders could do more to champion the voices of young people and allow them to be active participants in the decisions that are made about harmful commercial practices and health.

## Graphical Abstract

**Figure:**
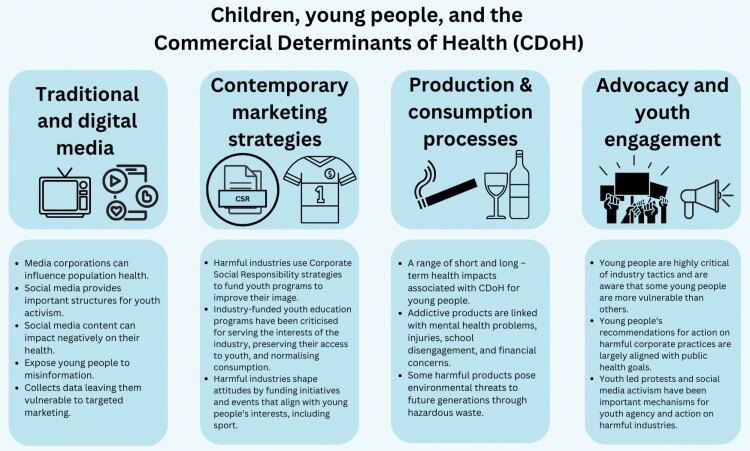

**CONTRIBUTION TO HEALTH PROMOTION**

- This article discusses the commercial determinants of health (CDoH) and the impact they have on young people.
- Research into the CDoH and young people has mostly focused on the influence of harmful industry advertising.
- Further research exploring broader tactics such as corporate social responsibility, is needed to understand how these strategies may feature and influence young people.
- Children and young people are powerful advocates for their own health and well-being and need to be given the opportunity to be a part of the decisions that are made to protect them from commercial tactics.

## DEVELOPMENTS IN COMMERCIAL DETERMINANTS OF HEALTH (CDoH) SCHOLARSHIP

Health is complex and political and requires public health and health promotion to be responsive to the range of factors that exist outside of the individual and that are vectors for poor health and well-being (Thomas and Daube, 2023; van Schalkwyk *et al.*, 2021a). Over the last decade, there has been an increased global focus on the impact of commercial practices on the health and well-being of populations. Commercial determinants of health (CDoH) scholarship—interlinked with the social (Marmot and Bell, 2019) and political (Kickbush, 2015) determinants of health—has helped to provide a new theoretical lens on health equity, moving beyond biomedical and behavioural models of health and disease (Freudenberg, 2023).

Until recently, those working in the CDoH have mostly investigated the range of tactics that health-harming industries (such as the tobacco, alcohol, ultra-processed food, fossil fuel and commercial gambling industries) use to promote their products, increase profits and prevent meaningful regulatory reforms that would protect health and well-being (Gilmore *et al.*, 2023, Lee, 2023). Sometimes called the ‘corporate playbook’, this has included tactics such as the impact of commercial marketing practices (Thomas *et al.*, 2023b); the influence of framing on how the public view responsibility for engagement with harmful products (Casswell, 2013; van Schalkwyk *et al.*, 2021b); corporate social responsibility (CSR) strategies (Mialon and McCambridge, 2018); industry influence over scientific research (Fabbri *et al.*, 2018; Vidaña-Perez *et al.*, 2023); the influence of political donations (Johnson and Livingstone, 2021); and lobbying (McCambridge *et al.*, 2014).

Researchers have recognized that the current research focus on the tobacco, alcohol and ultra-processed food industries has been understandable given the significant health and social burdens that they cause to communities (Gilmore *et al.*, 2023; Lacy‐Nichols *et al.*, 2023). However, newer industries such as vaping and online commercial gambling (Thomas *et al.*, 2023a), as well as the fossil fuel (Friel *et al.*, 2023) and firearm industries (Maani *et al.*, 2020), operate from a similar playbook and utilize a range of novel practices and promotions to diversify and expose a new generation of consumers to their products. Furthermore, recent frameworks have recognized that the CDoH encompasses much more than harmful industry practices (Lee *et al.*, 2022) and include the ‘*systems, practices, and pathways through which commercial actors drive health and equity*’ (Gilmore *et al.*, 2023, p. 1195). Scholarship has expanded to investigate the role that structure and agency may play in enabling and supporting, or challenging corporate practices (Lee *et al.*, 2022), including the role that governments play in intervening between these practices and health outcomes (Karreman *et al.*, 2023). These are important areas of focus for the public health and health promotion communities, given the emphasis in the Ottawa Charter for Health Promotion on equity and social justice, and the call in the Charter for policymakers to be ‘*aware of the health consequences of their decisions and to accept their responsibilities for health*’ (World Health Organization, 1986, p. 2). The focus on equity, agency and structure in recent CDoH frameworks also provides an important lens through which to investigate how corporate practices (and responses to these) may impact different population groups in different ways (Friel *et al.*, 2023). For example, in Sub-Saharan Africa, researchers have argued that investigations into the CDoH must move beyond harmful products, towards research that examines the impacts of extractive activities, urbanization, trade liberalization and commercialization in health systems (Loewenson *et al.*, 2022).

Just as with the social determinants of health, some populations are clearly more vulnerable to the impacts of corporate practices than others (McCarthy *et al.*, 2023a). The Lancet Commission on a Future for the World’s Children argued that the CDoH poses significant threats to the health and well-being of children and that mechanisms are needed to amplify their voices and skills to ensure a healthy and sustainable future for people and the planet (Clark *et al.*, 2020). Importantly, children and young people (subsequently referred to as young people) are not a homogenous group, and some young people are much more vulnerable than others, including First Nations and Indigenous children (Eisenkraft Klein and Shawanda, 2023), those from low socio-economic backgrounds (Mallol *et al.*, 2021) and girls (McCarthy *et al.*, 2023a).

To date, there have been few attempts to synthesize new areas of research, which consider the impact of the CDoH on the health of young people. The following article aims to address this gap by considering areas of CDoH research that may be particularly relevant to young people. We have focused on four areas: (i) the potential impact of traditional and digital media as a CDoH; (ii) the impact of contemporary marketing practices; (iii) the risks associated with corporate consumption and production practices; and (iv) the involvement of young people in advocacy initiatives as a way of responding to the CDoH.

## THE IMPACT OF TRADITIONAL AND DIGITAL MEDIA AS A CDoH

The commercial practices of the media and more recently social media industries are a significant CDoH (Zenone *et al.*, 2023). The activities of media corporations can influence population health and well-being (Brown and Witherspoon, 2002), including the role they play in framing health issues (Weishaar *et al.*, 2016). Social media sites have become central to how young people connect and socialize, express their creativity, become engaged in debates and discussions and form their identities (Juvalta *et al.*, 2023; Lyons *et al.*, 2023). For the health promotion and public health communities, they have also provided unique ways to connect with young people (Ferretti *et al.*, 2023; McCashin and Murphy, 2023; Taba *et al.*, 2023), and have been powerful platforms to motivate activism and advocacy around important health and social issues, such as the climate crisis (Boulianne *et al.*, 2020; Knupfer *et al.*, 2023). Social media platforms have provided important structures for strengthening young people’s agency and voice in counter-framing corporate and government narratives, and in inspiring collective global action on important health and social issues (Molder *et al.*, 2022).

However, there are also risks associated with the information that young people are exposed to through the media which may negatively impact their health and well-being—including the marketing of products that may be harmful to their health (Clark *et al.*, 2020; Soraghan *et al.*, 2023). Multiple studies have shown that the content that young people consume on social media platforms may contribute to heightened body image concerns (Choukas-Bradley *et al.*, 2022) and mental health issues (particularly for girls) (Twenge *et al.*, 2022). While new media platforms have enabled young people to easily access information about their health and well-being, it may also expose them to misinformation and disinformation based on a range of political ideologies that are difficult for them to navigate (Howard *et al.*, 2021; Juvalta *et al.*, 2023).

Data that is collected about young people via new media platforms is also central to helping industries target them with a range of products and ‘dark’ or covert marketing that may be harmful to their health (VicHealth, 2022). In particular, concerns have been raised about how social media may be used by harmful industries to target young people in lower-and-middle income countries (LMICs) (Bankole *et al.*, 2023). Exposure to tobacco content through traditional and social media platforms is associated with youth smoking behaviours (Nunez-Smith *et al.*, 2010), including lifetime use, past 30-day use and susceptibility to tobacco use among those who do not smoke (Donaldson *et al.*, 2022). Similarly, exposure to alcohol marketing across different media channels can increase the likelihood that a young person will initiate or increase their alcohol use (Anderson *et al.*, 2009). Newer social media platforms such as TikTok and Instagram have come under particular scrutiny for exposing young people to marketing for harmful products such as gambling, with a lack of regulatory compliance associated with new forms of marketing on these platforms, such as influencer promotions (McCarthy *et al.*, 2023b; Silver *et al.*, 2023).

Social media platforms have also enabled health-harming industries to create new mechanisms for directly engaging youth in their marketing activities, including using challenges, encouraging young people to tag friends and share content with social network members in posts and creating user-generated content (Bankole *et al.*, 2023; Brooks *et al.*, 2022). Young people are also exposed to promotions for products that they are not able to ‘legally’ consume. For example, while young people are not able to legally gamble in most countries until the age of 18 years, they report seeing marketing for gambling products on a range of social media platforms, including YouTube, Snapchat and Instagram (Pitt *et al.*, 2022a). These platforms have also enabled the industry to diversify from traditional young male markets to promote a range of female friendly campaigns on platforms such as TikTok designed to engage young women in gambling (McCarthy *et al.*, 2023b).

## THE IMPACT OF CONTEMPORARY MARKETING STRATEGIES ON YOUNG PEOPLE

To date much of the existing research into the impact of harmful industry practices on young people has focused on the impact of traditional forms of advertising such as commercial break advertising (Duke *et al.*, 2014; Pitt *et al.*, 2017; Pourmoradian *et al.*, 2020; Winter *et al.*, 2008). New definitions of marketing practices have included a much broader range of promotional strategies including promotions and incentives, and public relation activities such as CSR (Thomas *et al.*, 2023b). Here we provide several examples to highlight the importance of investigating how corporations may use CSR strategies to soften perceptions of risk, engage and feature young people and build brand loyalty. CSR is a marketing strategy that aims to improve a company’s image within the community, divert attention from the negative impacts of their products, influence policymaking and prevent regulatory reform (Thomas *et al.*, 2023a; Thomas *et al.*, 2023b). While CSR strategies are diverse, many have a specific focus on young people. Examples include British American Tobacco initiatives in Malawi to ‘eliminate’ child labour in tobacco growing practices (Otañez *et al.*, 2006); PepsiCo’s Refresh project which donated branded products to a range of youth initiatives including sporting programs (Dumbili and Odeigah, 2023); Nigeria Breweries running employment and skills training ‘empowerment’ programs for young people and women to respond to Sustainable Development Goal 8 relating to inclusive and sustainable economic growth (Otañez *et al.*, 2006); Shell Globals’ NXplorer program which helps young people *‘learn how to address the complex challenges faced by the world and to do something about them*’ (Shell Global, n.d.; Shell NXplorers, 2018); and a £10 million donation from the Betting and Gaming Council UK to fund gambling charity YGAM and treatment provider GamCare to deliver the ‘Young People’s Gambling Harm Prevention Programme’ (BGC, 2023).

While some of these initiatives might appear to be beneficial, Dorfman et al. (2012, p. 4) argue with specific reference to soda companies that CSR tactics are used to ‘*tout their concern for the health and well-being of youth while simultaneously cultivating brand loyalty*’. Analyses of tobacco industry documents found that the aim of youth education programs was not to reduce youth smoking rates but to ‘*serve the industry’s political needs by preventing effective tobacco control legislation, …preserving the industry’s access to youths, …and preserving the industry’s influence with policy makers*’ (Landman *et al.*, 2002, p. 917). Gambling and alcohol industry-funded youth education programs have also been criticized by researchers for serving their own interests by effectively diverting attention from the harmful aspects of the products and services they promote, and normalizing the consumption of these products (van Schalkwyk *et al.*, 2022a,b).

Corporations may also shape social and cultural attitudes and build corporate support among young people through funding initiatives and events that align with their interests. Historically, tobacco companies have sponsored music festivals and focused marketing at bars and nightclub events to embed their brand within youth culture, a tactic that has also been seen from e-cigarette brands (Tobacco Tactics, 2021; Truth Initiative, 2018). Alcohol companies have sponsored popular cultural events to attract the attention of young people (Yoon and Lam, 2013). The fossil fuel industry sponsored school-based maths and science education programs, including Exxon Mobile’s Education Alliance in the USA (Exxon, 2023), and Rio Tinto invested $2 million to provide access to quality early childhood education in the Pilbara region of Western Australia (RioTinto, 2022).

Finally, sports sponsorship remains a well-established and effective form of indirect marketing for harmful industries to influence the consumption behaviours of young people. Sports sponsorship has contributed to the increased uptake of smoking among young people (Tobacco Tactics, 2022), normalized gambling as a common part of sport (Pitt *et al.*, 2016; Djohari *et al.*, 2019; Pitt *et al.*, 2023) and has positively influenced young people’s attitudes towards unhealthy food and beverage sponsors (Cancer Council WA, 2023). BP Australia sponsored the Indigenous Nationals sporting event, including providing scholarships for student athletes to ‘*support their academic and athletic endeavours*’ (BP Australia, 2023). Importantly, research shows that young people may be less critical of the sponsorship activities of harmful industries as compared to their commercial activities, believing that sponsorship provides sporting teams and related organizations with important financial benefits (Pitt *et al.*, 2022b).

## THE RISKS OF CORPORATE PRODUCTION AND CONSUMPTION PROCESSES ON THE HEALTH OF YOUNG PEOPLE

There are a range of short- and long-term health impacts associated with the CDoH for young people. The impacts of addictive products such as alcohol and gambling include mental health problems (Ryan *et al.*, 2019), injuries (Chikritzhs and Livingston, 2021), disengagement from school (Ssewanyana and Bitanihirwe, 2018) and financial concerns (Livazović and Bojčić, 2019). Ultra-processed foods contribute to a range of non-communicable diseases among young people across the world (Nardocci *et al.*, 2019). Importantly while there have been significant reductions in the initiation and continuation of smoking in adolescents, rates of smoking in LMICs and among Indigenous young people remain comparatively high (Xi *et al.*, 2016; Heris *et al.*, 2020). Novel products such as vaping have also created a new range of health risks for young people (Jonas, 2022; Khan *et al.*, 2023), and the true extent of the health harms posed by these and other new products such as online gambling may not be seen for many years to come.

We also note that products such as tobacco and vaping pose environmental threats to future generations through increased hazardous wastes (Pourchez *et al.*, 2022), although these industries have utilized greenwashing and ‘environmental consciousness’ tactics to minimize the perceptions of these risks (Heley *et al.*, 2022). Young people are particularly at risk of the harms caused by extractive industries, due to instances of child labour, displacement from housing, exposure to pollution and waste, as well as the damage caused to the well-being of the planet (DFAT, 2017). The significant environmental impacts associated with greenhouse gas and carbon emissions will arguably jeopardize the ability of young people to live on a healthy planet (Clark *et al.*, 2020).

## YOUNG PEOPLE ARE SUPPORTIVE OF RESTRICTIONS ON THE PRACTICES OF HARMFUL CORPORATIONS

Much less research has investigated young people’s views about the CDoH (Soraghan *et al.*, 2023). Research with young people about the commercial determinants of the climate crisis (Arnot *et al.*, 2023c), gambling (Pitt *et al.*, 2022b), alcohol (Aiken *et al.*, 2018; Beccaria *et al.*, 2019), tobacco and vaping (MacGregor *et al.*, 2020; Pettigrew *et al.*, 2022) and harmful commercial marketing (Soraghan *et al.*, 2023) has documented that they are highly critical of industry tactics and aware of the influence of profit motives on their resistance to reforming their practices. Young people are skeptical that the ‘soft’ strategies used by harmful corporations will make a difference to health and social harms without strong government intervention (Arnot *et al.*, 2023b). They are also aware of health equity issues, and are concerned that some young people are more vulnerable to the tactics of harmful industries than others (Arnot *et al.*, 2023b; Soraghan *et al.*, 2023). However, there is still limited research that has explored the impact of the CDoH on specific groups of young people such as those from LMICs and Indigenous youth, and how they think these determinants should be addressed.

Young people’s recommendations for action are largely aligned with public health and health promotion goals. For example, in a New Zealand study, young people were asked what they would do about junk food marketing if they were Prime Minister for a day, with responses ranging from complete marketing bans, to changes to the content of marketing to ensure it was more honest and less deceptive (Signal *et al.*, 2019). A recent consultation with young people about the range of policies that could be used to counter harmful marketing also found that they were particularly concerned about the lack of regulation of marketing on digital platforms, including the presence of misleading marketing that created a perception that some harmful products were health promoting (Soraghan *et al.*, 2023). Similar recommendations have been made by young people in relation to gambling. Young people aged 11–17 years recommended reducing the accessibility and availability of gambling products, placing restrictions and bans on marketing, untangling the relationship between gambling and sport, making products safer and counteracting the positive messages about gambling (Pitt *et al.*, 2022b). Importantly, even when young people have a high social acceptance of novel products such as vapes, most are supportive of comprehensive government regulations of these products (Gorukanti *et al.*, 2017).

## YOUNG PEOPLE ARE INCREASINGLY INVOLVED IN ADVOCACY INITIATIVES TO TACKLE HARMFUL INDUSTRIES

Young people are increasingly involved in a range of advocacy initiatives demanding policy action on harmful industries. Examples include the work of young people in exposing the tobacco industry and more recently vaping tactics as part of the US-based (Truth Initiative, 2023), as well as action on unhealthy food marketing and access to healthy food through Bite Back in the UK (Bite Back, 2023). Another example, has been the powerful youth movement on climate justice, including Seed Mob, Australia’s first Indigenous-led youth group aiming to address climate injustices, which highlights the important role that First Nations youth can play in holding governments and industry accountable for a ‘*just and sustainable future*’ (Seed, n.d). Youth-led protests and social media activism have also been important mechanisms for youth agency and action on climate (Arnot *et al.*, 2023a), including a range of events and organizations inspired by Greta Thunberg’s activism like the now global School Strike 4 Climate (Boulianne *et al.*, 2020), Fridays for Future (Fridays For Future, 2023) and the UK founded Extinction Rebellion (Extinction Rebellion, n.d.); as well as gun control through the USA March For Our Lives student movement (Kelly, 2021; King, 2021; Zoller and Casteel, 2022). These forms of action may provide important opportunities for youth to be engaged in action on other CDoH.

## THE PUBLIC HEALTH AND HEALTH PROMOTION COMMUNITIES CAN STRENGTHEN YOUTH ENGAGEMENT IN THE CDoH

Addressing the CDoH and their impacts on young people must involve transformative approaches that not only listen to their views but engages them in decision making and policy action. We recommend three areas for focus:

1) CDoH researchers should collaborate with young people to investigate how a range of different corporate tactics, systems and structures may impact the health and well-being of young people. This involves considering the issues that young people see as the most important priorities in their own lives. While the impacts of new media platforms and mis/disinformation are areas of concern, we should also examine how new media platforms may increase the agency of young people to respond to the CDoH. Importantly, researchers should work with young people to define research agendas and areas for action, and should be mindful that the issues that young people prioritize may change over time.
2) While CDoH scholarship on young people is advancing, it is concerning that there has been so little research that has sought to directly engage young people (particularly those most at risk) in discussions about the CDoH, and the range of strategies that they think may be used to respond. There is much to be learned from non-governmental organizations working in these areas, who have engaged and supported youth to develop powerful youth-centred movements that have exposed industry tactics and argued for policy reform.
3) We should be careful about the potential ‘youthwashing’ of strategies to respond to the CDoH. This is where young people’s voices are used or platformed without initiatives that act on their key concerns or suggestions for action (Arnot *et al.*, 2023a; The Climate Reality Project, 2023). Mechanisms should be developed which engage young people in meaningful democratic participation in the decisions that are made about action on the CDoH, recognizing their agency as important stakeholders in the decisions that may impact their health and well-being.

There is much to be learned from young people about how we can respond more effectively to the CDoH. Advancing CDoH scholarship, advocacy and action must involve transformational change in how we engage and include young people to tackle the powerful vested interests of harmful and predatory industries.
